# Nanosecond pulsed electric field inhibits malignant melanoma growth by inducing the change of systemic immunity

**DOI:** 10.4317/medoral.22976

**Published:** 2019-07

**Authors:** Xingxing Zhang, Yunxia Zhang, Jian Chen, Yuyan Wu, Jue Zhang, Jing Wang

**Affiliations:** 1 M.S. School of Stomatology, Lanzhou University, 199 Donggang Western Road, Lanzhou Gansu 730000, China; 2 Graduate Student-M.S. School of Stomatology, Lanzhou University, 199 Donggang Western Road, Lanzhou Gansu 730000, China; 3 Prof. The First Hospital of Lanzhou University, 1 Donggang Western Road, Lanzhou Gansu 730000, China; 4 Graduate Student-M.S. School of Stomatology, Lanzhou University, 199 Donggang Western Road, Lanzhou Gansu 730000, China; 5 Associate professor. Academy for Advanced Interdisciplinary Studies, Peking University, Beijing, China; 6 Prof. School of Stomatology, Lanzhou University, 199 Donggang Western Road, Lanzhou Gansu 730000, China

## Abstract

**Background:**

Nanosecond pulsed electric fields (nsPEFs) showed an inhibitory effect on proliferation of malignant melanoma. In this study, the growth of melanoma were inhibited by changing the systemic immunity.

**Material and Methods:**

C57BL/6 mice with B16 malignant were exposed to 200 pulses of 100 ns duration, 30kV/cm. The mice were executed four days later. T lymphocyte has been extracted from spleen. Cell viability was evaluated by CCK-8 assay. CD3+CD4+ T cells, CD3+CD8+ T cells, regulatory T cells (Treg) and myeloid-derived suppressor cells (MDSC) were analyzed by flow cytometry. TNF-α, IL-2, IL-10, TGF-β, IFN– γ levels in supernatants were assessed by ELISA.

**Results:**

C57 malignant melanoma model were established successfully. After the treatment of nsPEFs(30 kV/cm 100 ns 200p), the numbers of T lymphocytes were increased.CD3+ CD4+ T cells changed from 48% to 51.2%;CD3+CD8+T lymphocytes increased from 39.6% to 40.4%.Treg cells reduced from 4.3% to 2.4%,MDSC decreased by 39.0% to 19.7% . In addition, the level of TNF-α, IL-2 were increased (*P*< 0.05) and the level of IL-10 were decreased (*P*<0.05) and the level of TGF-β and IFN-γ remained stable (*P*>0.05).

**Conclusions:**

Tumor growth can be effectively inhibited by nsPEFs *in vivo*, which activate targets of immune respones, accumulation of inflammatory cells and immune cytokines.

** Key words:**Nanosecond pulsed electric fields, melanoma, immune cytokines.

## Introduction

Cancer is the leading cause of death in developed countries and the second leading cause of death in developing countries (1). At present, medical treatment methods for tumors mainly include surgical resection, radiation therapy, chemotherapeutic drug treatment (2,3), traditional Chinese medicine treatment (4), and molecular immunotherapy (5), but these treatments have different degrees of limitations and side effects. Thus seeking for a new treatment is imminent.

The occurrence and development of tumors are closely related to the immune system, with the further development of the disease, tumor cells and immune cells competed in the host body. In the immune system, in order to prevent autoimmune reactions from occurring, some immune cells inhibit the immune response (6) such as MDSCs and regulatory T cells. Due to the presence of these cells, the balance of the immune system is maintained. IL-10 and TGF-β cytokines create an immune escape microenvironment during the development of tumors (7) , it exists long-term with tumor patients. In a word, the purpose of tumor immunotherapy is to stimulate or mobilize the body’s immune system to enhance the tumor microenvironment against tumor immunity, thereby controlling and killing tumor cells (8).

In recent years, nanosecond pulsed electric fields (nsPEFs) with short pulse duration, low energy density and non-thermal effects have caused a lot of attention. It can induce tumor cell apoptosis (9), anti-angiogenesis (10), thereby inhibiting tumor recurrence and metastasis. Cellular and animal models studies have confirmed the biological characteristics of tumor ablation, such as malignant melanoma (11), human osteosarcoma (12), pancreatic cancer (13). As a novel tumor therapy, it has shown a clinical perspective. But the specific relationship between systemic immune and nsPEFs is unclear. So in this study we will establish models of C57 malignant melanoma, and to explore the relationship between nsPEFs and systemic immunity. We will provide a new idea for tumor immunotherapy.

## Material and Methods

-Reagents

TNF-α, IL-2, TGF-β, IFN-γ, IL-10 ELISA kit (eBioscience, USA), CCK-8, lymphocyte separation medium, DMSO (Sigma, USA), DMEM, RPMI 1640, and FBS (Hyclone, USA), trypsin (Gibco, USA), Anti - Mouse CD3 - PE - Cy5, Anti - Mouse CD4 - PE, Anti - Mouse CD8 - FITC, Anti - Mouse CD4 - PE - Cy5, Anti - Mouse CD127 - Alexa Fluor 488, Anti - Mouse CD25 - PE, Anti - MouseCD11b - PE - Cyanine5, Anti-Mouse Ly-6G (Gr-1)-PE (eBioscience, USA).

-Instrument

Nanosecond pulse device was developed by Academy for Advanced Interdisciplinary Studies, PKU. Prototype parameters: 30 kV peak voltage, pulse width 100 ns, frequency of 1 Hz.

-Mice and cell 

C57 mice (female), 6-8 weeks, (18-20) g, were purchased from Charles river company, B16 malignant melanoma cell lines was provided by the basic medical college of Peking medical. Twenty C57 mice were inoculated subcutaneously with B16 melanoma cells on the back. The mice were divided into two groups: 10 tumor-bearing mice of nsPEF untreated group (control group) and 10 mice of nsPEF-treated group (therapy group). Each experiment repeated three times.

-The selection of experimental parameters

1x106/ml single cell suspension, take 0.2 mL cell subcutaneous inoculation into back after the mice was anesthesiaed by 1% pentobarbital sodium solution. the tumor (distribute in the subcutaneous nonmetastatic stage. CLARK IV: the Breslow thickness 1.51~3.00mm) were treated at 30 KV/cm 100 ns 300pulse, 200 pulse and 100 pulse, respectively. After four days, observe the ablation of the mice.

-T lymphocyte preparation

After the mice in each group were sacrificed, the spleen was taken aseptically, placed on a clean bench, rinsed with physiological saline and cut into pieces, ground with a sterile syringe needle, filtered through a 200-mesh sterile filter and the cell filtrate was collected and centrifuged at 1000r/min Centrifuged 5min, the supernatant was discarded in the sedimentation cells added 2mL red blood cell lysate mix, let stand 5min, the supernatant was centrifuged, the precipitate was mixed lymphocytes.

-CCK-8 detect the proliferation of T cells 

Executed the mice, separate the spleen, extract T lymphocytes to cultivate, adjust the cell density of 5 x 104/mL, vaccination in 96 - well cell culture plates, cultivate after 24 h, 48 h, to add the CCK-8 fluid, 37°C for 2 h incubation. 450 nm wavelength measured the optical density value of the cell. The proliferation rate = (OD nsPEFs – OD control)/OD control.

-Flow cytometry instrument to analysis CD3+CD4 +T cells, CD3+CD8+T cells, Treg and MDSC 

Adjust the density of lymphocyte cell to 1x105/mL take 100 uL spleen cell suspension, add antibody Anti - Mouse CD3 - PE - Cy5, Anti - Mouse CD4 - PE, Anti - Mouse CD8 - FITC detect CD3 +, CD4 + T cells and CD3 + CD8 + T cells, Anti - Mouse CD4 - PE - Cy Anti - Mouse CD127 - Alexa Fluor 488, Anti-Mouse CD25–PE to test Treg cells; Anti - Mouse CD11b - PE - Cyanine5, Anti-Mouse Ly-6G (Gr-1)-PE test MDSC cells, blending, avoid light, cultivate the cells in 4°C for 30 min, centrifugal, computer test.

-ELISA to analysis TNF-α, IFN-γ, IL-2, TGF-β, IL-10 

Pick the eyeball of mice for bloodletting, collect blood, 3000 r/min, the centrifugal 10 min, separate serum, test the sample. Room temperature balance ELISA kit for 30 min, put the standard and sample test enzyme label plate, 37°C for 2 h. Add Labeled antibody working liquid, 37°C incubate for 1 h; add HRP working liquid, 37°C incubate for 30 min; add TMB substrate solution, 20 min later add terminated liquid, finish the reaction, 450 nm standard wave length measure the optical density value of the tests. Use standard concentration and its OD value to make a standard curve, figure out the regression equation, put sample OD values into the equation, calculate the TNF-α, IFN-γ, IL-2, TGF-β, IL-10 levels.

-Statistical analysis 

All data were processed by Origin Professional 8.5 software. The statistical significance between the control and nsPEFs treatment groups was calculated by Student’s t-test. Significance was considered when *p*< 0.05.

## Results

-30 kV / cm 100 ns 200p nsPEFs inhibits tumor growth.

**Figure 1:**
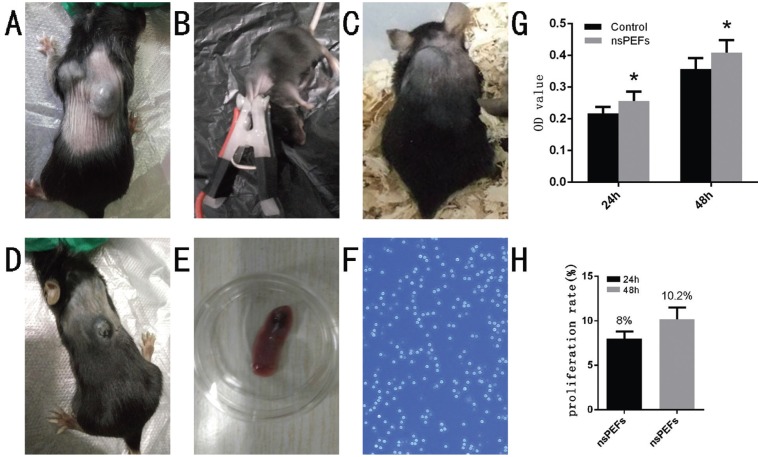
The change of C57BL/6 mice with B16 melanoma cells after treatment and extracted T lymphocytes from the spleen. 7 days after inoculation B16, the distribution of subcutaneous tumor can be found. Tumor-bearing mice treated by nsPEFs (Fig. 1B). Untreated tumor (Fig. 1A). The tumor with 30 kv,100 ns,100 pulses treated (Fig. 1C); the tumor with 30 kv,100 ns, 200 pulses treated (Fig. 1D). 4 days later, extracted T lymphocytes from the spleen (Fig. 1E). T cells morphology under a microscope(10x) (Fig. 1F). After treatment the survival rate of T lymphocytes increased in 24 hours and 48 hours compared with the untreated group (*p*< 0.05) (Fig. 1G,H).

7 days after inoculation B16, we found the distribution of subcutaneous tumor (Fig. 1A). The tumor-bearing mice were treated by 30 KV/cm 100 ns 100 pulse, 200 pulse, and 300 pulse respectively (Fig. 1B). the activity of mice was significantly reduced after treatment. The mice with 300 pulses were dead. In the 100 and 200 pulse treatment groups, the tumor size became narrowed after treatment, and the area of the tumor were more significantly reduced in 200 times than in the 100 pulse treatment groups (Fig. 1C,D), suggesting that the number of pulses was positively correlated with tumor ablation within the tolerance range of the mice. According to the efficacy of pulse therapy for melanoma. 30 kV/cm 100 ns 200p was chose as following-up parameters. 4 days after treatment of tumor-bearing mice, T lymphocytes were extracted from the spleen cells, cells grow in suspension (Fig. 1E,F). The survival rate of T lymphocytes after treatment of nsPEFs was tasted by CCK-8. As is shown (Fig. 1 G,H), after treatment the survival rate of T lymphocytes increased to 8% in 24 hours and 10.2% in 48 hours compared with the untreated group (*P*< 0.05). It shows that after nsPEFs therapy T lymphocyte proliferate and more immune cells were recruited. It suggested nsPEFs enhanced the cellular immune function.

-nsPEFs treatment of tumor-bearing mice enhanced cellular immune function and anti-tumor effects.

**Figure 2:**
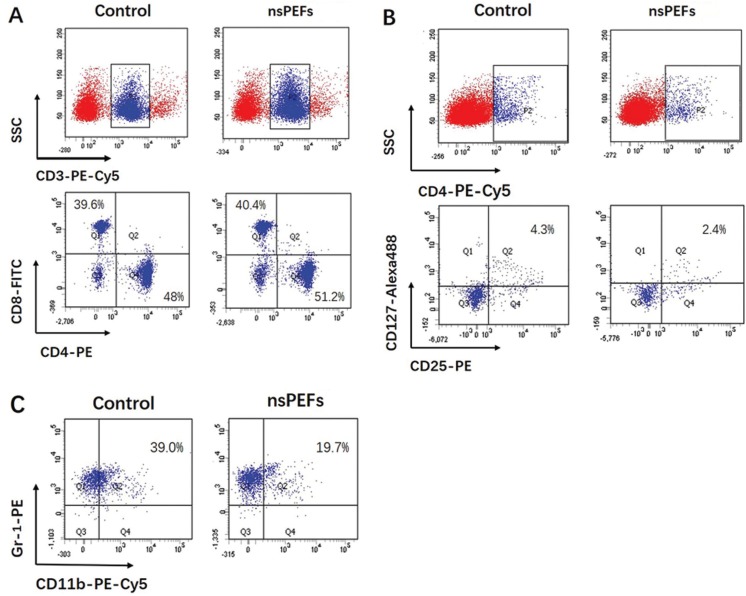
The flow cytometry analysis. Compared with the untreaed group, the content of CD3+CD4+T cells and CD3+CD8+T lymphocytes were increased (Fig. 2A). In contrast, the cells of treg and MDSC were reduced (Fig. 2B,C) (n≥3 independent experiments).

To further define change of immune system, the spleen was undertaken by flow cytometric. Compared with the untreated group, the content of CD3+CD4+T cells increased from 48% to 51.2%; the content of CD3+CD8+T lymphocytes increase from 39.6% to 40.4% (Fig. 2A). Treg cells reduce by 4.3% to 2.4% (Fig. 2B). MDSC reduced by 39.0% to 19.7% (Fig. 2C). The increased CD3+CD4+T can directly kill tumors, and these cells can cause the release of interferons and perforin to accelerate the destruction of tumor cells. The decrease in the percentage of immunosuppressed cells indicates that the immunosuppressive function of the body is weakened after stimulation, and the ability of immune cells to capture tumor cells is improved. In short, the results suggested that nsPEFs treatment of tumor-bearing mice enhanced cellular immune function and anti-tumor effects.

-The anti-tumor effect of nsPEFs-treated mice is related to the level of immune factors.

**Figure 3:**
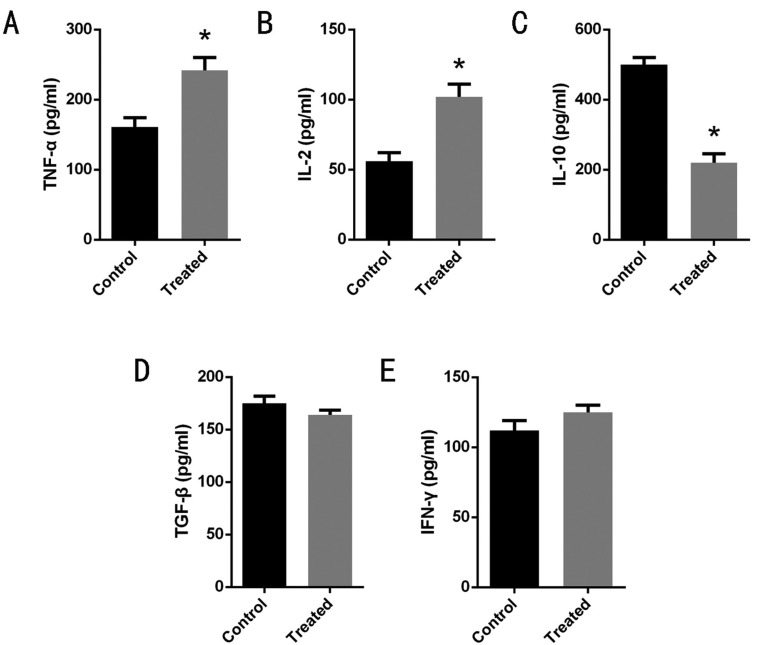
The results showed that after treatment IL-2 and TNF-α concentration increased (*p*< 0.05) (A,B); the level of IL-10 decreased (*P*< 0.05) (C). IFN-γ, TGF-β had a rising trend, but no statistical difference (*P*>0.05) (D,E). All data are presented as means± standard deviations (n≥3 independent experiments).

To investigate the mechanisms of T cell activation, we analysis the immune factor of tumor-bearing mice(non-treatment group VS nsPEFs treatment group). As is shown on, after the treatment, the level of IL-2 and TNF-α increased (*P* < 0.05) (Fig. 3A,B). the content of IL-10 reduced (*P* < 0.05) (Fig. 3C). IFN-γ, TGF-β increased without statistical difference (*P* > 0.05) (Fig. 3D,E). IL-2, TNF-α, and IFN-γ immune factors enhanced the function of tumor-killing cells such as CD3+CD4+T and NK cells. The experimental results suggested that the anti-tumor effect of nsPEFs treatment is related to the level of immune factors, and the function of immune cells is enhanced with the change of immune factors. All the data indicated the imbalance of cytokines plays a role of anti-tumor effect in mice.

## Discussion

The malignant melanoma model as a classic model is closely related to the development of immunotherapy, so the model of this study is based on the C57BL/6 mouse malignant melanoma xenograft model. This article successfully combines the two hot spots of current malignant melanoma treatment. Pulse upper limit based on non-thermal effect is 300 ns (14). We used a pulse width of 100 ns, a field strength of 30 Kv/cm, and a pulsed electric field of 100 pulse, 200 pulse and 300 pulse with a frequency of 1 Hz as a variable for the treatment of malignant melanoma xenografts. We found that the tumor growth can be effectively inhibited with the treatment( 30 kV/cm ,100 ns, 200p nsPEFs). Studies have shown that the volume and weight of fibrosarcoma in mice were reduced by 60% after 8 days of treatment with nsPEFs (15). The nsPEFs(43 k V/cm, 20 ns, 200 p nsPEFs) treatment of basal cell carcinoma could gain therapeutic results (16). These results are consistent with our research. Of course, different parameters of pulsed electric fields are different on antitumor effects of varied tumors (17-18). Previous adoptive immunotherapy kills tumor cells and kills normal cells that have a common antigen with tumor cells. But we utilized nsPEFs to trigger the autoimmune system immunity of the body, effectively avoiding this problem.

T lymphocyte is the key of cancer therapy (19-20). To evaluate this effect, in our research, the CCK-8 analysis showed that nsPEFs can directly kill tumor cells by promoting T lymphocyte proliferation. T lymphocyte mainly contains cytotoxic CD8+T cells and CD4+T cells. Antitumor immunity involves the priming of both CD4+T and CD8+T. To evaluate the effect, we used flow cytometry for detection. The results confirmed that the application of nsPEFs can enhance the cytotoxic effect of immune cell to target tumor cell. Analogously, nuccitelli et al found that it can stimulate specific immune response of tumor cells and inhibit the growth of tumors (21).

After confirming the effect of T lymphocyte, we evaluated Treg and MDSC. They are immunosuppressive cells that allow tumor cells to escape immune surveillance (22-23). Patients with long-term tumor-bearing survival have a lower number of Treg cells and a higher number of cytotoxic T cells (24). Our study showed that conformed that 30 kV/cm, 100 ns 200pulse, 1 Hz electric field treatment inhibited Treg and MDSC. This suggests that nsPEFs may inhibit immune escape in the treated host except immune cell. In addition,the systemic immunity contains all kinds of cytokines. Tumor necrosis factor-alpha can cause hemorrhage and necrosis of tumor cells, directly kill tumor cells, cause local inflammatory reaction, and promote the killing ability of monocyte macrophages. IL-2 and IFN-γ in mouse serum are mainly produced by activated Th1 cells, it can activate NK cells to induce T cell proliferation, Th0 to differentiate into Th1 cells, and then produce more cytokines, IL-10 and TGF-β create a microenvironment for immune escape during the development of tumors (25-26). To assessment the change of cytokines affected by nsPEFs we used ELISA kit to perform. The results demonstrated that after the treatment, the level of IL-2 and TNF-α increased, the content of IL -10 reduced. In short, 30 kV/cm, 100 ns 200pulse, 1 Hz electric field treatment can stimulate the changes of the content of anti-tumor immune factors and play an important role in assisting immune cells.

NsPEFs therapy for melanoma is similar to immunotherapy which reinfuse immune cells to host or a large immune response caused by surgical resection. Both of them can cause the body produce an immune response. According to report (27), CD4+T cells can be detected in both nsPEFs treated tumor and untreated secondary transplantation tumor. The result further confirmed that nsPEFs can activate tumor immune responses. According to this study, we can guess that nsPEFs stimulates the immune system indirectly. The body can not rapidly clear the early apoptotic cells induced by nsPEFs. It may has not been eliminated by phagocytes and perforin granzymes. These necrotic cells are recognized by the memory cells and carry out antigen presentation to activate the body’s immune response. However, this hypothesis needs further study. Whether nsPEFs change the immune system directly or not? It still needs further study.

Our current understanding of immunology was largely defined in laboratory mice, partly because they are inbred and genetically homogeneous, can be genetically manipulated, allow kinetic tissue analyses to be carried out from the onset of disease, and permit the use of tractable disease models. Laboratory mice reflect relevant aspects of the human immune system, but, it is subject to the constraints of pulse electrodes and can only be used to treat small tumors. There is no more research on larger metastases. So, it has the limitations of an animal experimental model in its use in humans notability. The first-in-human safety trial of nanoelectroablation indicated that this new therapy is safe and may offer a fast and scarless alternative to the current standard of care for small basal cell carcinoma tumors (28). However, the nanosecond pulse is also a category of high voltage. Although it can induce tumor cell apoptosis, on the other hand, it has potential harm to the organism, which should be highly valued by us, and the corresponding protective measures should be taken. It is hoped that this study can provide new ideas for the combination of physical therapy with immunotherapy in the future and provide a new experimental basis for the clinical application of nsPEFs to treat tumor.
